# Effect of a Virtual Reality–Enhanced Exercise and Education Intervention on Patient Engagement and Learning in Cardiac Rehabilitation: Randomized Controlled Trial

**DOI:** 10.2196/23882

**Published:** 2021-04-15

**Authors:** Victoria Gulick, Daniel Graves, Shannon Ames, Pavitra Parimala Krishnamani

**Affiliations:** 1 Information Services & Technologies Jefferson Health Philadelphia, PA United States; 2 Rehabilitation Medicine Thomas Jefferson University Philadelphia, PA United States; 3 Department of Emergency Medicine Baylor College of Medicine Houston, TX United States

**Keywords:** virtual reality, VR, cardiac rehabilitation, patient experience, patient education, outpatient therapy, exercise

## Abstract

**Background:**

Cardiac rehabilitation (CR) is clinically proven to reduce morbidity and mortality; however, many eligible patients do not enroll in treatment. Furthermore, many enrolled patients do not complete their full course of treatment. This is greatly influenced by socioeconomic factors but is also because of patients’ lack of understanding of the importance of their care and a lack of motivation to maintain attendance.

**Objective:**

This study aims to explore the potential benefits of virtual reality (VR) walking trails within CR treatment, specifically with regard to patient knowledge retention, satisfaction with treatment, and the overall attendance of treatment sessions.

**Methods:**

New CR patients were enrolled and randomized on a rolling basis to either the control group or intervention group. Intervention patients completed their time on the treadmill with VR walking trails, which included audio-recorded education, whereas control patients completed the standard of care therapy. Both groups were assisted by nursing staff for all treatment sessions. Primary outcomes were determined by assessing 6-minute walk test improvement. In addition, secondary outcomes of patients’ cardiac knowledge and satisfaction were assessed via a computer-based questionnaire; patient adherence to the recommended number of sessions was also monitored. Cardiac knowledge assessment included a prerehabilitation education quiz, and the same quiz was repeated at patients’ final visit and again at the 2-month follow-up. The satisfaction questionnaire was completed at the final visit.

**Results:**

Between January 2018 and May 2019, 72 patients were enrolled—41 in the intervention group and 31 in the control group. On the basis of the results of the prerehabilitation and postrehabilitation 6-minute walk test, no significant differences were observed between the intervention and control groups (*P*=.64). No statistical differences were observed between groups in terms of education (*P*=.86) or satisfaction (*P*=.32) at any time point. The control group had statistically more favorable rates of attendance, as determined by the risk group comparison (*P*=.02) and the comparison of the rates for completing the minimum number of sessions (*P*=.046), but no correlation was observed between the study group and reasons for ending treatment.

**Conclusions:**

Although no improvements were seen in the VR intervention group over the control group, it is worth noting that limitations in the study design may have influenced these outcomes, not the medium itself. Furthermore, the qualitative information suggests that patients may have indeed enjoyed their experience with VR, even though quantitative satisfaction data did not capture this. Further considerations for how and when VR should be applied to CR are suggested in this paper.

**Trial Registration:**

ClinicalTrials.gov NCT03945201; https://clinicaltrials.gov/ct2/show/NCT03945201

## Introduction

### Background

Cardiac rehabilitation (CR) is an underutilized but vital part of recovery following a cardiac event. However, many patients do not attend or complete the recommended number of treatment sessions. According to the Million Hearts Initiative, only approximately one-third of all eligible patients participate in CR treatment [1]. Previous studies have shown that CR decreases “morbidity and mortality, improves clinical outcomes, enhances psychological recovery, and decreases the risk for secondary cardiac events” [1] and that a 12-week program can reduce cardiovascular risk factors for over a year post treatment [2]. A myriad of social, economic, and cultural factors contributes to the low percentage of patients who participate in CR [3]. However, even among patients who can attend and afford sessions, many do not complete their entire course of treatment. Research has shown that a lack of motivation [4] and a lack of understanding [5] contribute to this behavior.

A fundamental part of outpatient CR treatment is access to education. At many CR centers, such as the Jefferson Health Methodist CR facility, nursing staff conduct periodic lectures while patients exercise. However, because of the number of patients or timing of sessions, most educational materials are distributed in paper format, either on bulletin boards or as handouts.

To address the lack of interest in treatment and difficulties in providing access to patient education, we decided to pursue the use of digital technologies as an alternative, potentially more engaging way of conveying information to patients. This was founded on the work of the Digital Innovation and Consumer Experience (DICE) Group. The DICE Group used technology to advance the future of health care and education at Jefferson Health. Originally formed within the DICE Group, the XR (extended reality) Lab works to create a collaborative environment in which health care meets XR technology. XR is an all-encompassing term for augmented, virtual, and mixed reality technology. This team studies the potential benefits of XR technology for education and patient care and has pursued projects using XR, including patient experiences during bone marrow biopsies, advanced cardiac life support training for resident physicians, anatomy education for medical students, in-clinic vestibular therapy exercises, and nursing education for patient falls prevention.

Virtual reality (VR) has previously been employed in areas of health care, ranging from preoperative patient anxiety [6] to poststroke patient education [7], but it has rarely been used in conjunction with CR. Most notably, several studies have examined VR in combination with stage 3 CR when patients continue their physical activity at home. One survey revealed that 64% of patients felt motivated to continue exercising with the VR application after primary program completion [8]. At the time of this project’s development, there was a lack of conclusive evidence about VR’s effect on stage 2 outpatient rehabilitation.

### Objectives

This study intends to address the lack of interest and comprehension exhibited by many CR patients, which may contribute to incomplete treatment. Education was directly incorporated into patient time on the treadmill, thereby increasing the number of patient exposures to cardiac health information. In addition, digital technologies, including VR, were explored as an alternative way to increase patient engagement with care. Overall, this study aims to evaluate whether employing a VR program that incorporated patient education could increase patients’ motivation, understanding, and adherence to CR treatment.

## Methods

### Study Design

The study procedures were approved by the Jefferson Institutional Review Board (IRB) in December 2017. Beginning in January 2018, participants were selected from patients enrolled in CR at the Jefferson Health Methodist CR program on a rolling basis. Sample size calculations indicated that 68 patients were required to achieve a target power of 90%. Patients were randomized via the Google random number generator, limited to a range of 1-2—1 signifying the control group and 2 signifying the intervention group. To prevent patient bias, the consenting process did not include VR terminology or any reference to VR walking trails.

### Participants

Participants were recruited between January 2018 and May 2019. All patients were emailed or mailed a copy of the consent form to review with the rest of their welcome materials before visiting the facility. At patients’ first visit, the facility staff stratified patients according to their risk: low, moderate, or high, as per facility standard guidelines. Initially, only moderate-risk patients were enrolled, but this was subsequently expanded to all risk-level stratifications (following approval from the IRB in July 2018) to increase enrollment numbers. Patients were deemed eligible to participate after agreement of 2 study staff members (Textbox 1). If the staff determined that a patient was eligible to participate, they were consented in a private room on the same day.

**Eligibility criteria for participation in trial.**

**Eligibility criteria**

- Aged 18 years or older
- Ability to use a treadmill independent of aid (eg, walker and cane)
- Medically safe to use a treadmill for 15 minutes
- Ability to understand English
- Ability to give their own consent

At this time, patients were scheduled according to their assigned study group—control patients at 9 AM and 11 AM sessions and intervention patients at 8 AM, 10 AM, and 1:30 PM sessions. Patients who could not attend during one of their randomized time slots were excluded from the study and scheduled at their preferred time. Normally, the facility accommodates 10 patients per session. To account for space limitations, VR participants were limited to 5 per session to allow for adequate time with 1 of the 2 VR systems. The other slots in sessions that already contained 5 VR participants were filled by nonparticipants.

Admission to the study continued until 72 patients were enrolled: 31 in the control group and 41 in the intervention group. Patients in both the control and intervention groups completed a 6-minute walk test (6MWT) at their introductory visit to establish a baseline.

### Intervention

#### Study Procedures

Immediately after consenting to participate, patients were given a 5-question education quiz (Textbox 2) to establish their baseline cardiac education level.

**Education questions for pretest, posttest, and 2-month follow-up test.**

**Education quiz**

- How often should you exercise?
- What are the most important things to consider when grocery shopping for heart-healthy foods?
- How often should you take your blood pressure medication?
- What are the symptoms of heart failure?
- What type of medicine is used to control your cholesterol?

For their first 3 visits, participants spent approximately 5 minutes on the treadmill, increasing at intervals of 1 minute if deemed appropriate by the staff. The remainder of each session took place on other types of exercise equipment, as per standard of care. Beginning at their fourth visit, patients were allowed to use the treadmill for up to 15 minutes at each visit. Patients walked at a fixed walking speed as self-paced treadmills are not used at the facility. In addition, research has shown that participants using fixed walking speeds had increased stride length compared with participants on self-paced treadmills [9].

All patients were scheduled to complete between 18 and 36 sessions, as decided by their care team and insurance provider. Typically, low-risk patients were encouraged to complete between 18 and 24 sessions, moderate-risk patients were encouraged to complete between 24 and 30 sessions, and high-risk patients were encouraged to complete between 30 and 36 sessions. CR staff recommended that patients complete the maximum number of possible sessions for their risk level, but the final number was decided by the patient.

Patients were contacted again 2 months after their final CR treatment. It was decided to follow up with patients at 2 months postrehabilitation, as, on average, patients were expected to complete approximately 2 months of treatment. This phone call was designed to follow up on patients’ health following treatment and to assess their retention of education by repeating the 5-question education test conducted at their initial and final visits. Previous studies have shown that the use of VR increases the retention of education in students learning complex anatomical concepts [10]. Patients who could not be reached by phone were contacted via email. Those who stopped attending CR sessions before their determined treatment length were also contacted to follow-up and debrief from the study. At least three attempts were made to contact each participant before considering them lost to follow-up.

#### Control

Patients allocated to the control group received standard of care CR, with the option for additional time on the treadmill, up to 15 minutes, beginning at session 4. Standard of care CR includes completing time on 4 types of exercise equipment, including treadmills, stationary bikes, ellipticals, and hand rowing machines.

Another standard part of CR is education on heart health. Patients are exposed to educational materials to help them understand how to better care for their heart during recovery and to give them tools to continue improving their health in the future. Control patients received standard of care education through handouts, bulletin board displays, and periodic group lectures.

Finally, participants were debriefed at their last scheduled session and informed about the VR intervention. At this time, they were able to try the VR walking trails if desired.

#### VR Intervention

Participants in the VR intervention group completed the standard of care CR for the majority of their exercise, except for time spent on the treadmill. While using the treadmill, they used the Bionautica Trails system [11], a VR walking trails platform designed and produced by Plas.md [12]. Nursing staff logged each patient with their unique study ID and allowed them to select 1 of 6 walking trails, including 5 nature trails of different themes and 1 outer space–themed trail (Multimedia Appendix 1).

At each log-in, patients had the option to continue from their saved place on a trail from the previous session or select a new one. As patients walked, visual tokens popped up on screen, and when the patient *walked through* them, a randomly chosen piece of cardiac education information was triggered to start (Multimedia Appendix 2). These were programmed to filter through all 109 audio files before repeating. These audio files were taken directly from the handouts and CR textbooks used by the nursing staff. A 2015 study showed that patients who received both auditory and visual cardiac education were more likely to participate in their treatment [13].

### Materials

**Figure 1:**
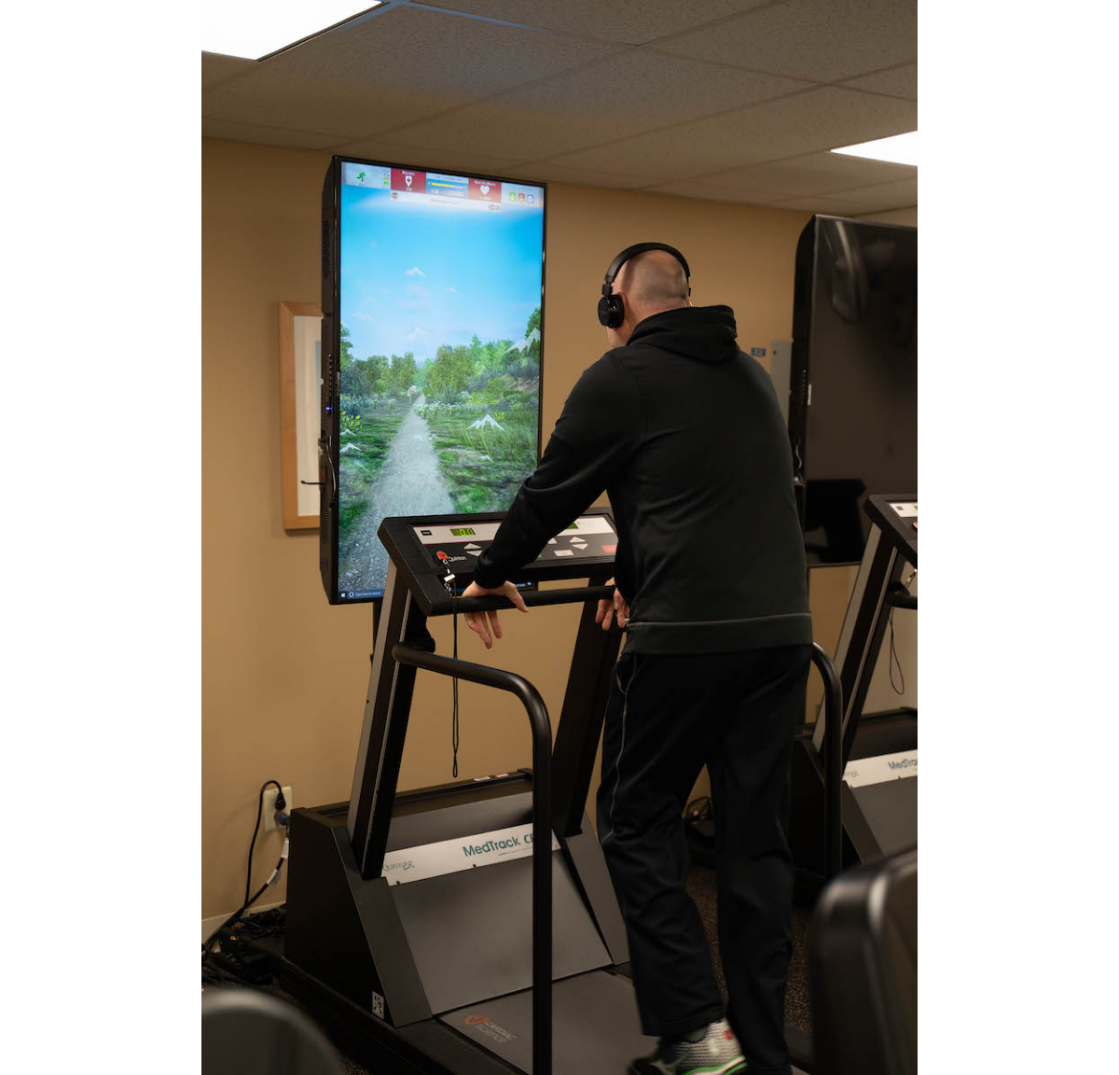
Vertically oriented television screen in front of a treadmill showing an example of Bionautica Trails.

The VR platform, Bionautica Trails, was set up as a high-definition flat-screen television, oriented vertically on a stand in front of 1 of 2 treadmills designated for the study (Figure 1). This system was selected over head-mounted VR options for the safety of the patient. Each of the 2 systems was run on an individual computer hard drive and connected to a Bluetooth keyboard with a built-in trackpad. For the purposes of the study, the Bionautica Trails platform was not updated and remained frozen in the version initially used to maintain consistency between participant experience regardless of when they started treatment.

The educational audio files each intervention group patient heard during their treatment were recorded in a deidentified cloud-based server, via Microsoft Azure, to track individual exposure to education and frequency. Patients used on-ear, wireless headphones, connected to the walking trails via Bluetooth, to better immerse themselves and allow other patients to continue exercising without distraction. These were noise reducing but not noise canceling to ensure that patients could communicate properly with nursing staff. Headphones were disinfected using AF3 antibacterial wipes provided by the facility, as per standard infectious disease guidelines.

### Instruments

#### Survey Tool

At their last scheduled session, after finishing their exercise, patients completed a web-based survey housed on the Qualtrics platform [14], a Health Insurance Portability and Accountability Act–compliant survey tool, through an anonymous link. This survey was designed by the authors to address the patient population at the Jefferson Health Methodist CR facility. Patients completed the survey using their study ID number rather than any identifying information, and only 1 member of the study staff had access to responses for analysis to protect patient privacy. The key variables measured in this self-report questionnaire were knowledge retention, patient satisfaction, and engagement (Multimedia Appendix 3).

#### Knowledge Retention

Knowledge retention for this study was defined as the maintenance of cardiac health information over time. Patients were asked a series of 5 questions (Textbox 2) at 3 time points: their first visit, last visit, and 2-months post treatment. These questions were identified by nursing staff as being vital to patients’ recovery and common areas of confusion for patients. Knowledge retention was scored out of 5 for all 3 time points, with each question worth 1 point.

#### Patient Satisfaction

Patient satisfaction is contentment with the care received and the overall experience of a health care interaction. Patients completed 6 questions rated on a 4-point Likert-type scale (extremely dissatisfied=1; dissatisfied=2; satisfied=3; extremely satisfied=4). Each question covered a different subtopic of satisfaction and was scored individually.

#### Engagement

Patient engagement is defined as an intrinsic interest in and participation in care. Patients completed 3 questions on their engagement, rating each feature on a scale of 1-10 wherein 1 was “not at all engaged” and 10 was “extremely engaged.”

### Statistical Analysis

This study was powered by an increase in the distance traveled during the 6MWT. The 6MWT is considered the functional walk test of choice for assessing clinical improvement in cardiorespiratory patients [15]. To estimate the expected improvement, equivalent data from patients with chronic obstructive pulmonary disease were used, which suggested an average improvement of 446 m (SD 82 m) for moderate-risk patients [16]. It was considered reasonable that the experimental group would achieve an additional 15% increase in the distance walked over that of the control group or an additional 67 m in 6 minutes because of their increased motivation and education from the intervention. Power analysis showed that to detect a between-group difference in the 6MWT at a power level of 0.90 and an α of .05, 34 participants were required in each group.

The 6MWT analysis was conducted using a *t* test allowing unequal variances; the Levene test for the equality of variances was used to determine equal variances for 6MWT improvement. For knowledge retention, a 2-way multivariate analysis of variance (MANOVA) test was used to compare improvement between groups at each of the following time intervals: pretest to posttest, posttest to follow-up, and pretest to follow-up. The MANOVA root included calculations for the Pillai trace statistic, the Wilks λ statistic, the Hotelling trace criterion, and the Roy largest test.

## Results

### Patient Demographics

**Figure 2:**
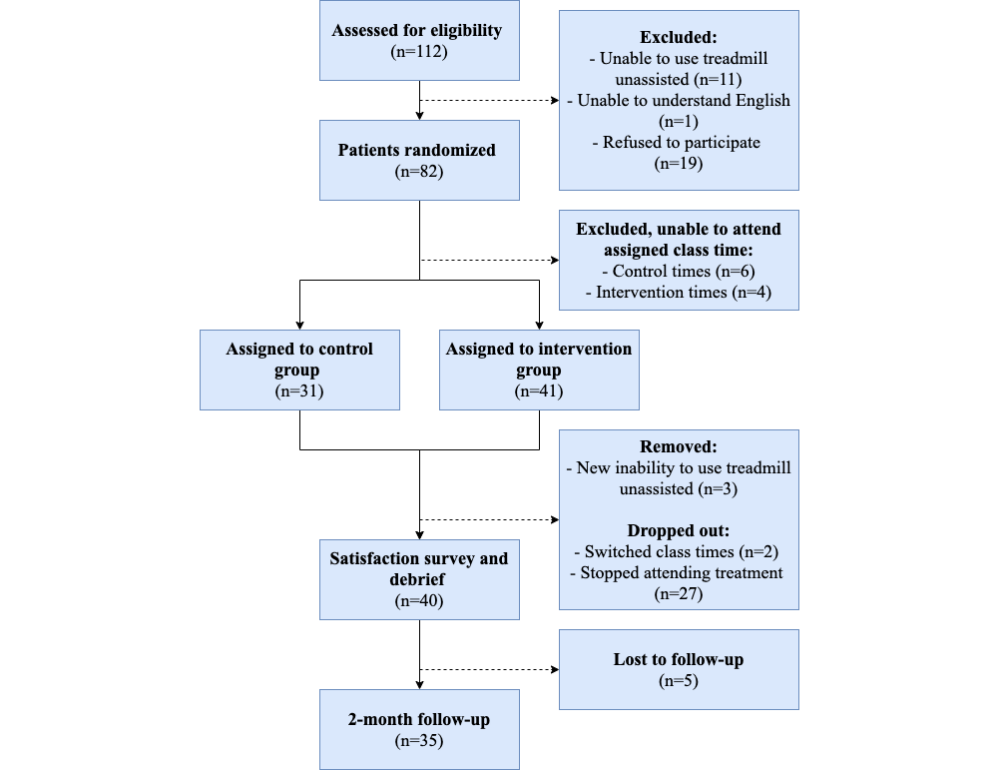
Flow diagram of research design and patient completion status.

Between January 2018 and May 2019, 72 patients (male: n=52; female: n=20; age: range 32-81 years) were enrolled in the study to either the control (n=31) or intervention (n=41) group (Table 1). Of these, 3 participants were excluded from the study because of an inability to continue exercise on the treadmill, and 35 participants (control: n=19; intervention: n=16) completed all stages of the study (Figure 2).

**Table 1:** Patient demographic data (N=72).

Characteristic		Patients
Age (years), mean (SD)		61 (9.9)
**Age (years), n (%)**		
	<40	2 (3)
	40-49	7 (10)
	50-59	19 (26)
	60-69	32 (44)
	70-79	11 (15)
	≥80	1 (1)
**Sex, n (%)**		
	Male	52 (72)
	Female	20 (28)
**Race, n (%)**		
	Asian	2 (3)
	Black (non-Hispanic)	21 (29)
	Hispanic	3 (4)
	White (non-Hispanic)	45 (63)
	Other	1 (1)

### Outcomes

#### 6MWT

All patients (N=72) completed the 6MWT during their first treatment session. All patients who completed the program (n=34) completed a postrehabilitation 6-minute walk test (post-6MWT) as well. In addition, patients who chose to terminate their program early by notifying staff completed the post-6MWT (n=6) at their last scheduled session. All patients who did not finish treatment and did not notify staff before terminating their participation did not complete the post-6MWT (n=32). For these patients, their final session’s distance walked was used to estimate their post-6MWT results for primary outcome improvement evaluation.

A blinded interim analysis was conducted in January 2019, which showed no statistical difference between improved distance walked relative to the 6MWT (*P*=.60; 95% CI −196.42 to 329.12)*.* As no increased risk was seen in either group, it was decided to continue with the original intended sample size for complete analysis of the primary and secondary outcomes. At the final review, patients in both groups showed improvement between pre- and post-6MWT distances. The control group improved by an average of 298 feet and intervention group by 340 feet, without significance between groups (*P*=.64; 95% CI −224.14 to 139.52).

#### Knowledge Retention

**Figure 3:**
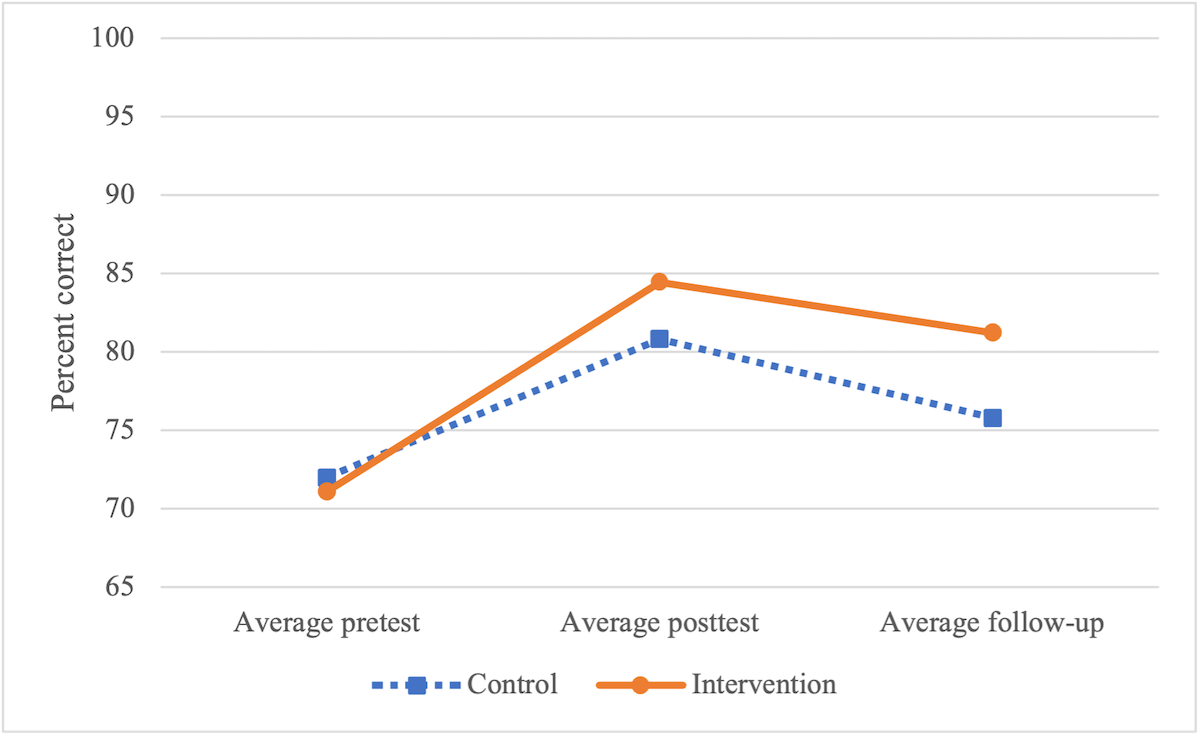
Average education test scores for pretest, posttest, and 2-month follow-up.

Patient knowledge retention was evaluated at 3 time points: at the first visit, at the last visit, and at 2 months post treatment. Patients were asked to answer 5 multiple-choice questions at all time points (Textbox 2). No significant difference was seen between groups for baseline (*P*=.88; 95% CI −0.53 to 0.62), final visit (*P*=.56; 95% CI −0.79 to 0.43), or follow-up (*P*=.50; 95% CI −1.09 to 0.54) quizzes (Figure 3). Overall, patients in the intervention group improved by an average of 10.1% between pretest and follow-up compared with 3.8% for the control group (*P*=.86; 95% CI 0.71-0.59). Patients in the intervention group received an average of 115 pieces of audio education from a bank of 109 audio files, at an average of every 100 seconds of walk time.

#### Patient Satisfaction and Engagement

At their final visit, the patients completed a survey on their satisfaction. Patients were asked to rate their satisfaction with the education they received and their CR experience, based on several subtopics (Table 2). Participants from both groups responded positively to their treatment, and no significant difference was observed between the groups. Patients were also asked to rate their enjoyment of time spent on the treadmill (Table 3) and their engagement with this portion of their treatment (Multimedia Appendix 4). Additional questions were asked about patient engagement overall and specific to the education they received to help understand the breakdown of patient engagement. For education specific engagement, 56% (22/39) of patients selected a rating of 10 out of 10. For overall engagement, 70% (28/40) of patients selected a rating of 10 out of 10. Again, no significant differences were observed between the groups.

**Table 2:** Satisfaction survey questions (control: n=21 and intervention: n=19).

Question			Control, mean score (SD)		Intervention, mean score (SD)		*P* value
**How satisfied are you with your CR^a^ experience?**							
	How satisfied are you with your CR experience overall?	3.7 (0.7)		3.9 (0.3)		.32	
	How satisfied are you with your time spent on the treadmill?	3.5 (0.7)		3.6 (0.5)		.45	
	How satisfied are you with your interactions with staff?	3.9 (0.6)		4.0 (0.0)		.35	
**How satisfied are you with your CR education?**							
	How satisfied are you with the delivery of your CR education?	3.8 (0.4)		3.5 (0.5)		.06	
	How satisfied are you with the personalization of your CR education?	3.7 (0.5)		3.5 (0.5)		.23	
	How satisfied are you with the clarity of information?	3.8 (0.4)		3.6 (0.5)		.23	

^a^CR: cardiac rehabilitation.

**Table 3:** Patients’ response to “During most of your treatment sessions, you were allowed to use the treadmill for up to 15 minutes. Did you enjoy the time spent on the treadmill?”

Answer choice	Control, n (%)	Intervention, n (%)
“Yes, I would have enjoyed even more than the time I was allowed to spend on the treadmill.”	6 (29)	5 (26)
“Yes, I enjoyed the time I spent on the treadmill. I did not need more than 15 minutes. It was the right amount of time for me.”	13 (62)	14 (74)
“No, I did not enjoy it, so I did not use the full 15 minutes.”	0 (0)	0 (0)
“No, I did not enjoy the treadmill. I do not enjoy that form of exercise.”	1 (5)	0 (0)
“I am undecided.”	1 (5)	0 (0)

Patients in the intervention group also completed the VR-specific questions (Table 4). Most participants responded negatively (“disagree”) to negative statements and positively (“agree”) to positive statements. Notably, the statement “I wanted to spend longer on the treadmill” resulted in a mix of positive, negative, and undecided responses.

**Table 4:** Virtual reality–specific satisfaction (n=19).

Question—“Because of the virtual reality walking trails...”	Disagree, n (%)	Undecided, n (%)	Agree, n (%)
“I looked forward to treatment sessions more.”	1 (5)	5 (26)	13 (68)
“I enjoyed my treatment sessions more.”	1 (5)	0 (0)	18 (95)
“I felt more engaged in my treatment.”	1 (5)	1 (5)	17 (89)
“I wanted to spend longer on the treadmill.”	7 (37)	6 (32)	6 (32)
“I gained a better understanding of my cardiac health.”	2 (11)	0 (0)	17 (89)
“I did not see any impact on my treatment.”	13 (68)	1 (5)	5 (26)
“I was unwantedly distracted from my treatment.”	16 (84)	1 (5)	2 (11)
“I did not enjoy how I received my education.”	17 (89)	0 (0)	2 (11)
“I felt isolated from the rest of my class.”	15 (79)	0 (0)	4 (21)
“I dreaded my time on the treadmill.”	15 (79)	1 (5)	3 (16)

#### Attendance

In addition, total patient attendance was recorded by group (Table 5). Each risk stratification had a different recommended number of sessions, but the Jefferson Health Methodist CR facility requires all patients to complete at least 18 sessions to be considered finished with treatment. As such, attendance was evaluated through both the recommended number of sessions and the completion of at least 18 sessions. Of the patients who were removed from or dropped out of the study (n=32), 4 control and 3 intervention patients continued with or returned to CR treatment after study dropout and were thus omitted from the attendance totals.

The comparison of attendance between each risk group showed that the control group had significantly higher completion rates (*P*=.02; 95% CI 0.04-0.53), as did the comparison of the rates for completing 18 sessions (*P*=.046; 95% CI 0.00-0.47). However, patients who stopped attending treatment sessions were asked to explain their choice, and no causal relationship was seen between poor attendance and VR walking trails. The reasons for ending treatment sessions early were mainly associated with other health issues (n=7), such as chronic pain, or a need to return to work (n=8); of those who needed to return to work, 2 patients switched to a new class time and 1 patient later returned to treatment but had already been debriefed about the study. Of the 29 patients who stopped attending treatment sessions, 11 left without explanation and could not be reached for follow-up.

**Table 5:** Patient attendance by completion of minimum required sessions and by recommended number of sessions.

Attendance		Control, n (%)	Intervention, n (%)
**Completion of the minimum required treatment sessions**			
	Fewer than the required 18 treatment sessions completed	5 (19)	16 (42)
	18 or more treatment sessions completed	22 (81)	22 (58)
**Completion of the recommended number of treatment sessions**			
	Fewer than the recommended number of treatment sessions	8 (30)	22 (58)
	Met or exceeded the recommended number of treatment sessions	19 (70)	16 (42)

## Discussion

### Principal Findings

This randomized controlled trial (RCT) focused on improving patients’ experiences during CR treatment through an alternative VR solution. To the best of our knowledge, this is the first RCT focusing on VR for knowledge retention and motivation conducted in stage 2 outpatient rehabilitation. Previous studies related to VR’s usage in stage 3 CR positively affected patient motivation compared with controls. The results demonstrated that there were no statistically significant outcomes, although this study provides valuable insights into the application of a virtual tool in outpatient CR.

### Primary Outcome

It was hypothesized that VR-enhanced outpatient CR would be superior to conventional outpatient CR in improving 6MWT distances. This was chosen as the primary outcome as a means of standardization, as it is a common measure of improvement for patients undergoing CR. However, the results indicated that the intervention and control groups had comparable improvements in their 6MWT distances. These results may be related to limitations of the 6MWT, including its use of patient-initiated walking speed rather than the manual speed of the treadmills, and the fact that patients completed exercises on an average of 4 different types of equipment per session. These factors may have limited the 6MWT’s ability to capture the value that VR can bring to a CR environment.

Furthermore, 44% (32/72) of patients did not complete a post-6MWT, severely limiting the sample size. We decided to use these patients’ final treadmill sessions and extrapolate their distance walked in 6 minutes. However, as these were from manually set speeds, this extrapolation did not capture patient effort during the 6MWT, which may have been impacted by using Bionautica Trails.

### Secondary Outcomes

#### Patient Engagement

Patient engagement was chosen as a secondary outcome because of evidence that VR has motivational benefits for patients in other rehabilitation settings, including during motor rehabilitation [17] and stroke rehabilitation [18].

Subjective measures of engagement were assessed through the patient survey, but an attempt was made to evaluate an objective measure of engagement based on the time spent on the treadmill. Patients were told they had up to 15 minutes of time on the treadmill, a duration chosen by the nursing care team to ensure patient safety and also fit within regularly scheduled sessions. By allowing patients to choose how long to spend on the treadmill, the hope was that they would spend time on the treadmill proportional to their interest level, based on the understanding that when participants are intrinsically motivated, they adhere better to exercise regimens [19].

This inference proved to be incorrect, as 62.5% (45/72) of patients, regardless of group, used the full 15 minutes at 60% of sessions or more. Those who did not use the full time typically cited coexisting health issues, such as arthritic joints, as reasoning for completing less than the full 15 minutes. Most patients likely chose to complete the full 15 minutes because many patients, particularly those of this age group, hear health care providers’ instructions as recommendations rather than as choices [20]. To improve how engagement can be better assessed in a similar outpatient environment, future studies should explore how the phrasing of patient instructions affects their choices.

#### Patient Satisfaction

It was unlikely that the intervention would improve overall satisfaction, as at baseline, the Jefferson Health Methodist CR center already had incredibly high satisfaction scores. This has been largely attributed to the incredible care of the nursing staff. Patients reported that “the positive attitude and support of the staff was THE most critical factor” and the “staff took time to check-in regularly on physical and emotional health - Program clearly help[ed] me regain and improve my overall health not just cardiac.”

Although no differences were seen between groups in terms of satisfaction, it is worth noting that many patients responded positively to Bionautica Trails. One participant reported, “It took me out of the room for a little bit; I really imagined hiking somewhere with my brothers,” and others highlighted how “the walking trails made the walking experience enjoyable.” The few patients who did not respond positively cited that they felt isolated from the social environment of the center or felt that the trails became repetitive. Although VR may not satisfy all patients, it should be mentioned that Bionautica Trails is still in use at the Jefferson Health Methodist CR center. Patients in both the treatment and maintenance programs use it regularly, and the nursing staff appreciate having the virtual trails as a tool for maintaining the focus of patients who need more attention.

#### Knowledge Retention

Previous research suggests that VR has the potential to improve patient comprehension of information [21] and long-term knowledge retention in students [10,22], possibly through spatial learning [22] or by increasing motivation to learn [23]. The knowledge retention test was limited to only 5 questions to minimize the amount of time added to required sessions, but it appears that this may have been too few questions to show a distinction between groups.

In addition, the questions themselves may have been too simplistic; at their initial visit, 86% (62/72) of patients knew that statins are used to treat cholesterol and 94% (68/72) knew how often blood pressure medication should be taken. It is difficult to show a significant change when there is little room for improvement. A more challenging set of questions, including a wider variety of complexities, should be used in future studies measuring the educational value of VR in this setting.

#### Attendance

Patient completion of the recommended number of rehabilitation sessions indicated statistical significance in favor of the control group. One possible explanation for this is that the enjoyment of VR could not overcome the considerable social and economic factors that patients face, which contribute to low attendance [3]. Patients who stopped attending treatment sessions were asked to explain their choice, and no causal relationship was seen between poor attendance and virtual walking trails. One of the most common reasons for dropping out was the need to return to work (n=8). Patients would often say that they had to miss work to attend treatment or that their job would only cover so much sick leave. In addition, although only 1 patient reported dropping out of treatment because of their insurance, several patients completed only 2 days per week of treatment—rather than the recommended 3—to keep copay costs at a minimum.

Given the complexity of balancing work, finances, and treatment schedules, VR did not work as a tool to increase patient attendance at CR sessions. However, it highlights the importance of introducing digital tools in conjunction with practical strategies that address patients’ needs.

### Patient Safety

In total, 3 patients were excluded from the study because of a new inability to use the treadmill. Two of these patients experienced a change in their ability to use the treadmill related to their preexisting health conditions. The third patient discontinued participation in the trial because of self-described motion sickness, also referred to in the literature as “cybersickness” [24]. During the course of the study, 41 patients collectively completed 139.6 hours of virtual walking trails—almost 6 full days of content—and the third patient was the only patient to report any adverse side effects. Although cybersickness should still be considered when conducting research, the exceptionally low incidence rate in this study supports the use of VR regardless of age or previous experience using VR technology.

### Blinding

Although not directly correlated to outcomes, it is worth discussing the difficulties faced when designing RCTs using VR. The nature of VR means that neither patients nor onsite study staff could be blinded to the study arm. Instead, the terms “virtual reality” and “virtual walking trails” were never mentioned during the consent process to blind patients to the study goal and limit response bias. Therefore, the patients were debriefed on their participation at their final treatment visit. At that time, those in the control group were offered the chance to try the walking trails. Most participants did not wish to try them when approached at the end of their last session, likely because they had already completed their workout and did not wish to spend additional time on the treadmill. However, post-6MWTs were occasionally conducted 1-2 sessions before the patient’s expected end date, typically when multiple patients were expected to finish on the same day or if there was concern that the patient might not return for the final treatment session. Those who completed their post-6MWT early often chose to use Bionautica Trails for their final time(s) on the treadmill. It is unclear why this trend occurred, but based on qualitative responses from patients, it supports positive patient engagement with the trails. One such patient, when asked if they would have enjoyed the VR walking trails during regular treatment sessions, commented:

I feel like I missed out. It made the time pass so fast. It was great. It’s different when you’re looking at a picture, it makes it go quick [sic]

### Future Research

The use of VR in rehabilitation is a growing field, and there are numerous potential avenues for further research. Adaptations to this project could include expanding Bionautica Trails to other types of exercise equipment, such as a recumbent bike, to assess how continuity between exercise equipment and increased exposure would affect results. It would also be valuable to conduct a similar trial with a more robust education component, particularly expanding the number of questions and including variable question complexity.

Beyond this specific trial, research should continue to explore the gamification of treatment. In the version of Bionautica Trails used for this study, each token that patients *walked through* contributed to an overall score. These scores remained private, but the creation of a social network scoreboard could potentially increase the motivation to continue walking [25]. Furthermore, the newest version of Bionautica Trails includes a story mode that may encourage repeated use and promote behavioral changes through narrative communication [26]. Although it may still not increase attendance rates of this particular patient population, given the socioeconomic factors discussed previously, this addition may address patients’ complaints about the repetitive nature of the trails. In addition, the use of a story mechanic lends itself particularly well to at-home or stage 3 treatment for maintaining user engagement without the external motivators inherent to the gym setting. Further research is needed to determine the most appropriate setting and population for using VR in the CR treatment process.

### Conclusions

Given the importance of CR treatment, it is vital to continue studying methods of improving patient access to care and patient experience while in care. Despite the need for CR, many patients do not complete treatment. Although this particular tool did not show statistically significant improvements in outcomes, it provided anecdotal positive reactions from patients and their health care providers. The limitations of the study design likely contributed to the final results. Further studies are needed to determine whether virtual walking trails are worth implementing in stage 2 CR, particularly accounting for the numerous socioeconomic factors that influence patient access to care. VR tools such as Bionautica Trails should continue to be explored to improve the overall patient experience.

## Appendix

Multimedia Appendix 1 Additional images of Bionautica Trails.

Multimedia Appendix 2 Video demonstrating Bionautica Trails in action.

Multimedia Appendix 3 Complete patient survey.

Multimedia Appendix 4 Patient response to the question “On a scale of 1 to 10, how engaged were you with your cardiac rehabilitation experience?”.

Multimedia Appendix 5 CONSORT-eHEALTH checklist (V 1.6.1).

## Abbreviations

6MWT: 6-minute walk test
CR: cardiac rehabilitation
DICE: Digital Innovation and Consumer Experience
IRB: Institutional Review Board
MANOVA: multivariate analysis of variance
Post-6MWT: postrehabilitation 6-minute walk test
RCT: randomized controlled trial
VR: virtual reality
XR: extended reality
