# Supplementary material for: Effect of a Virtual Reality–Enhanced Exercise and Education Intervention on Patient Engagement and Learning in Cardiac Rehabilitation: Randomized Controlled Trial
Source: J Med Internet Res. 2021 Apr 15;23(4):e23882. doi: 10.2196/23882 (PMC8085751; doi:10.2196/23882)

This is a Multimedia Appendix to a full manuscript published in the J Med Internet Res.
For full copyright and citation information see http://dx.doi.org/10.2196/jmir.23882

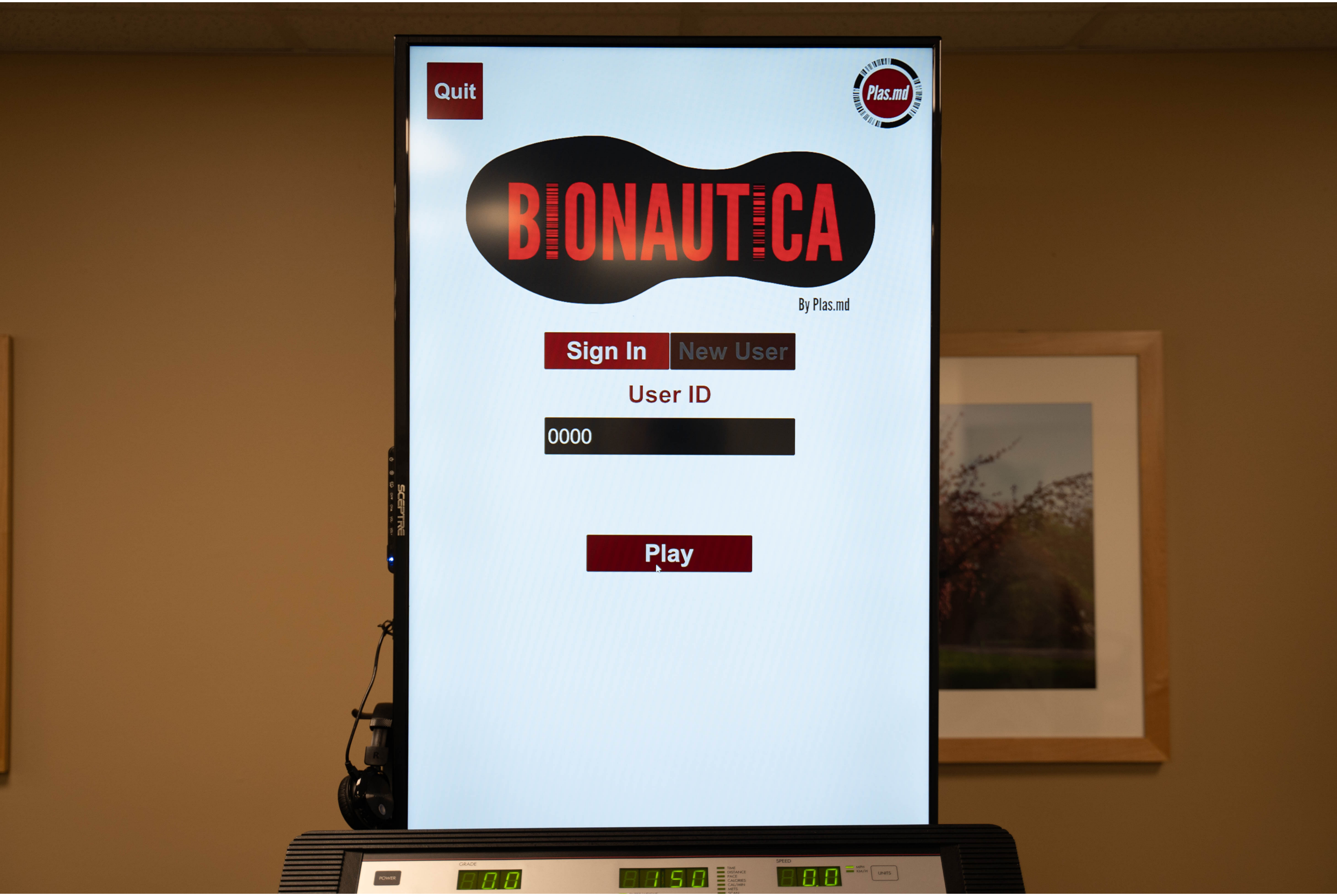

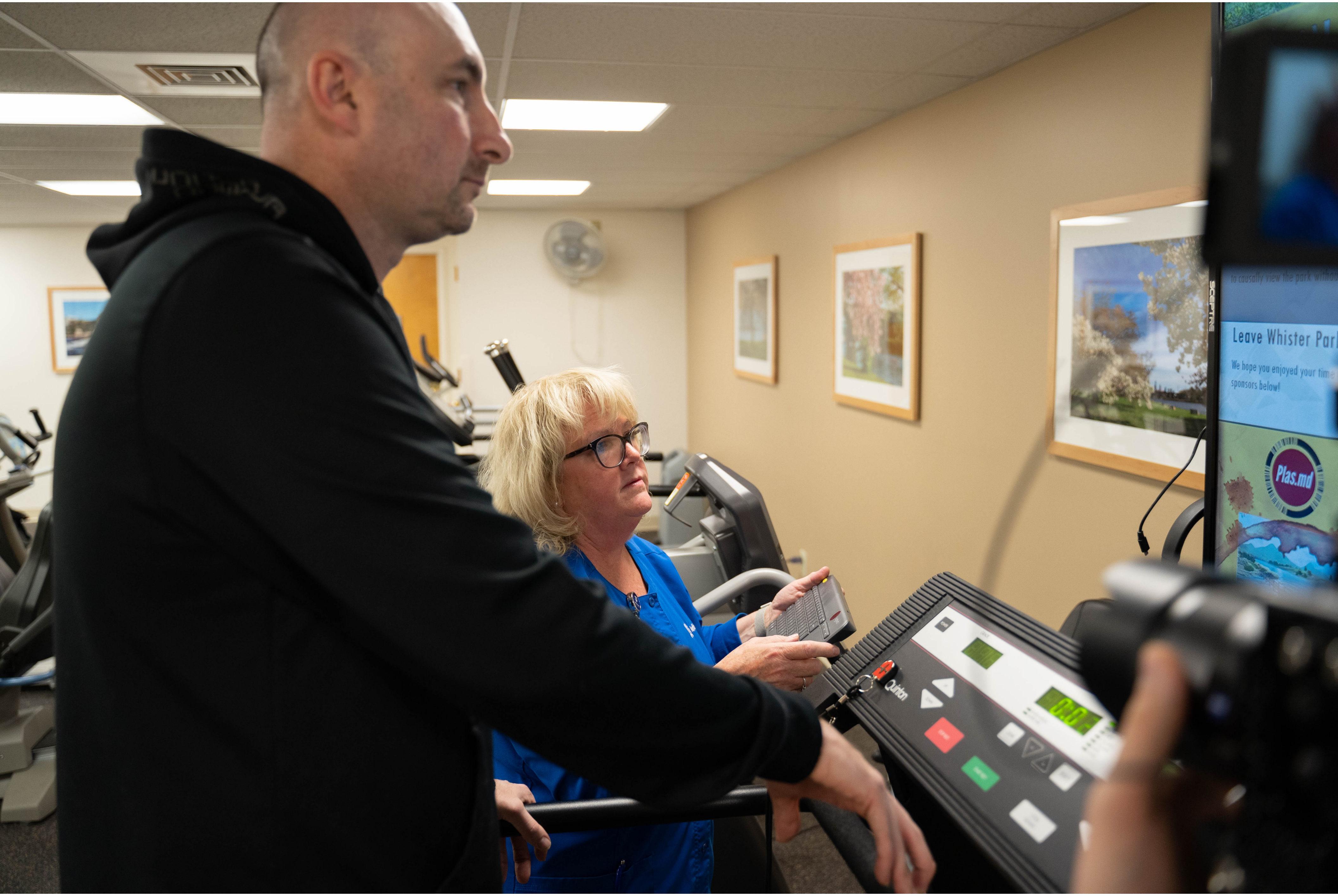
Leave Whister Par
We hope you enjoyed your time
sponsors below!
Plas.md
Quinton

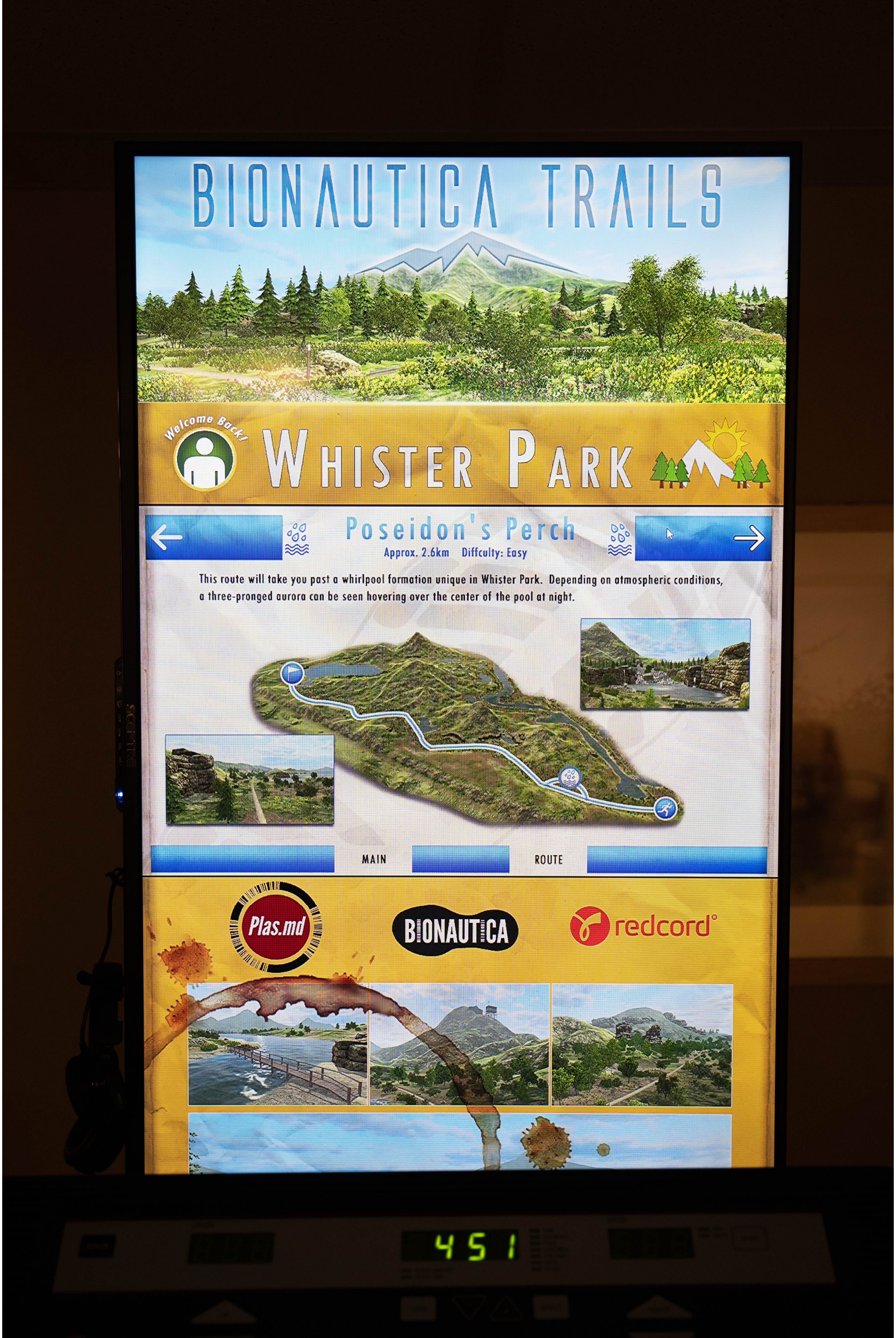
BIONAUTICA TRAILS
Welcome Back!
WHISTER PARK
Poseidon's Perch
Approx. 2.6km Diffculty: Easy
This route will take you past a whirlpool formation unique in Whister Park. Depending on atmospheric conditions, a three-pronged aurora can be seen hovering over the center of the pool at night.
MAIN
ROUTE
Plas.md
BIONAUTICA
redcord
451

BIONAUTICA TRAILS

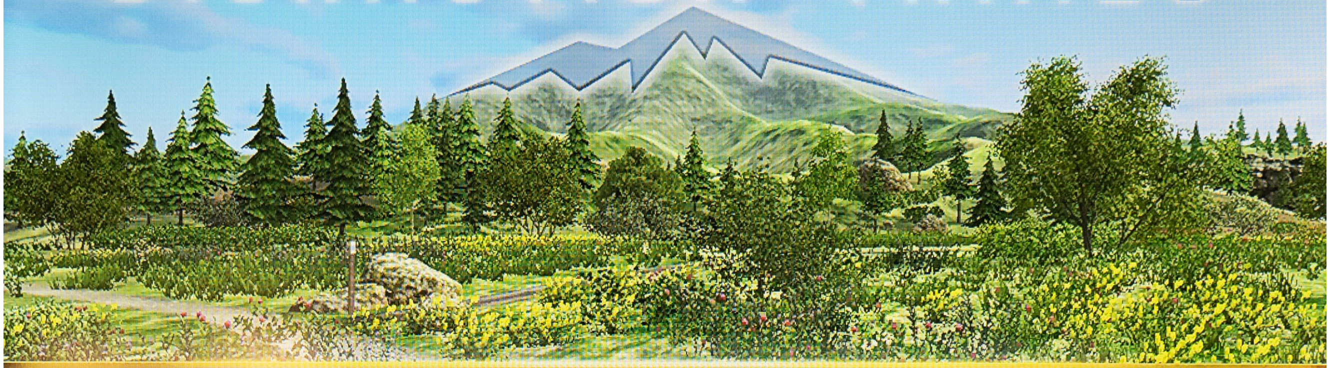

Welcome Back!

WHISTER PARK

The Void

Approx. Unknown Diffculty: Unknown

A group of hikers in the 1950's reported being whisked off of the trail they were following into a otherworldly landscape. Although their stories could not be verified, the urban legend of this mysterious trail was born.

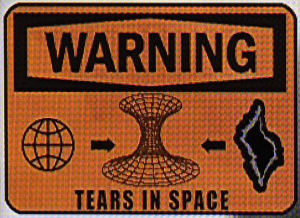

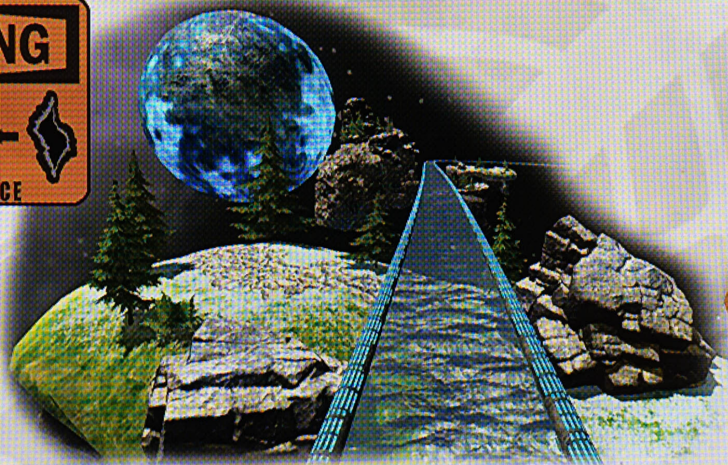

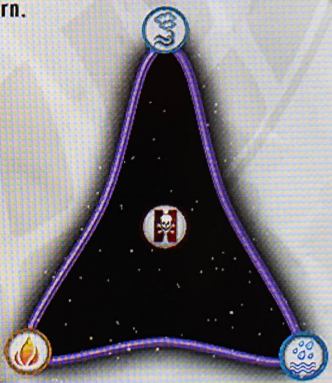

MAIN ROUTE

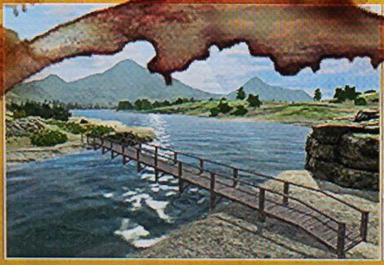

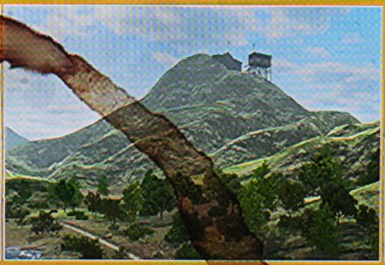

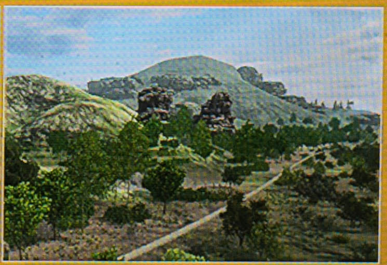

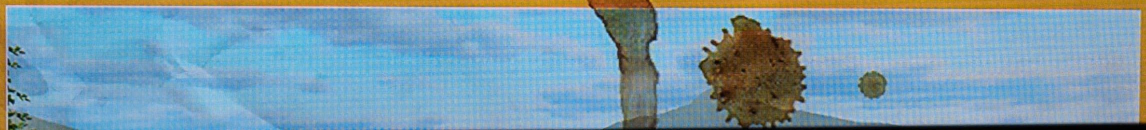

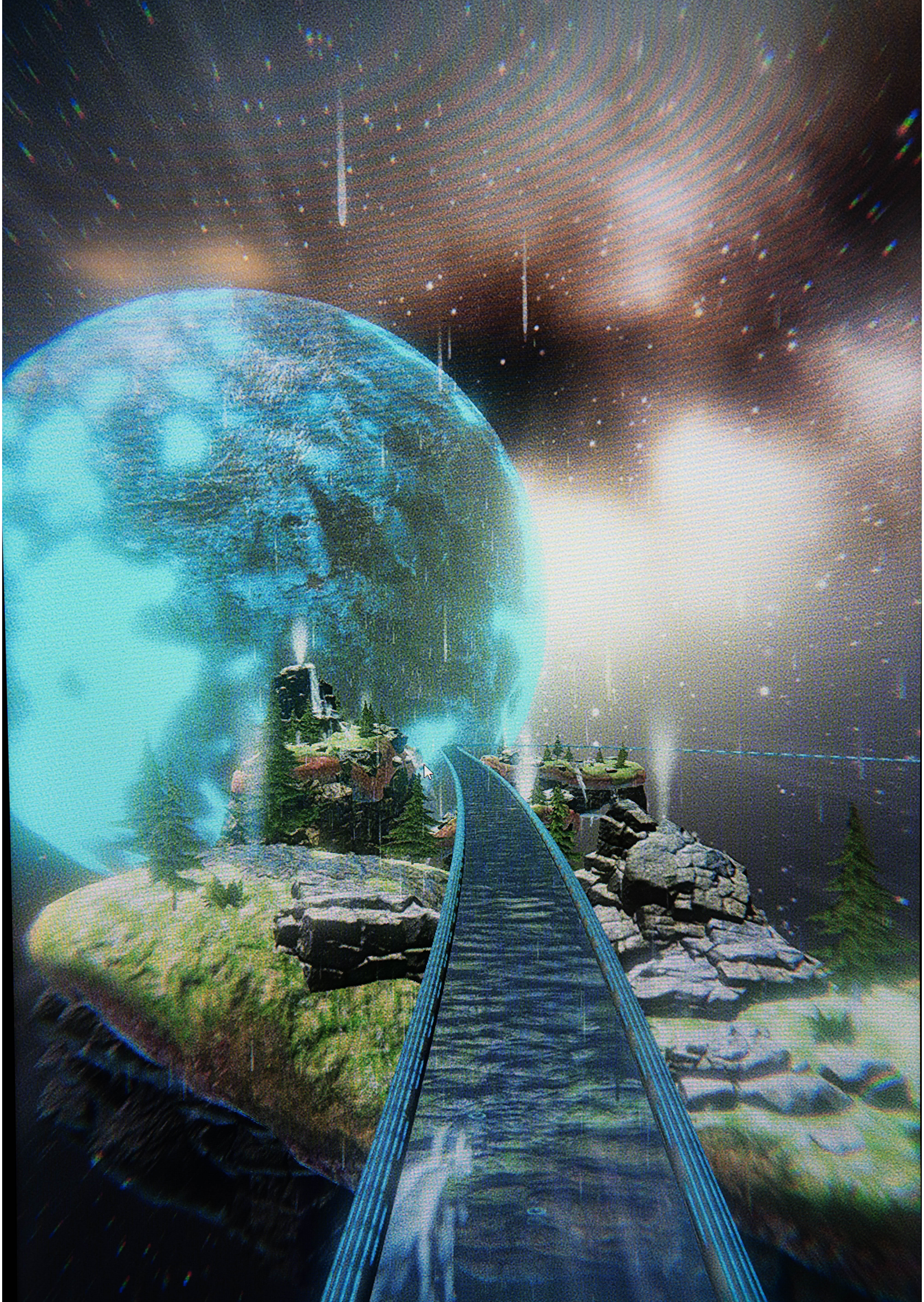

Supplement: Multimedia Appendix 1 [file jmir_v23i4e23882_app1.pdf]
