# Supplementary material for: Effect of a Virtual Reality–Enhanced Exercise and Education Intervention on Patient Engagement and Learning in Cardiac Rehabilitation: Randomized Controlled Trial
Source: J Med Internet Res. 2021 Apr 15;23(4):e23882. doi: 10.2196/23882 (PMC8085751; doi:10.2196/23882)
Supplement: Multimedia Appendix 3 [file jmir_v23i4e23882_app3.pdf]

This is a Multimedia Appendix to a full manuscript published in the J Med Internet Res. For full copyright and citation information see http://dx.doi.org/10.2196/jmir.23882

Cardiac Rehabilitation Exit Survey

Start of Block: ID

Q1.1 Please enter your study ID number.

____

Q1.2 During what session did you complete cardiac rehabilitation?

- 8 am
- 9 am
- 10 am
- 11 am
- 1:30 pm

End of Block: ID

Start of Block: Satisfaction

Q2.1 How satisfied are you with your cardiac rehabilitation experience?

	Extremely Dissatisfied	Dissatisfied	Satisfied	Extremely Satisfied
Overall	○	○	○	○
Time spent on treadmill	○	○	○	○
Interactions with staff	○	○	○	○

Q2.2 During most of your treatment sessions, you were allowed to use the treadmill for up to 15 minutes. Did you enjoy the time spent on the treadmill?

- ○ Yes, I would have enjoyed even more than the time I was allowed to spend on the treadmill.
- ○ Yes, I enjoyed the time I spent on the treadmill. I did not need more than 15 minutes. It was the right amount of time for me.
- ○ No, I did not enjoy it so I did not use the full 15 minutes.
- ○ No, I did not enjoy the treadmill. I do not enjoy that form of exercise.
- ○ I am undecided.

Q2.3 On a scale of 1 to 10, how engaged were you with your cardiac rehabilitation experience?

	1 = Not at all engaged	2	3	4	5	6	7	8	9	10 = Extremely engaged
Overall	○	○	○	○	○	○	○	○	○	○
Time on the treadmill	○	○	○	○	○	○	○	○	○	○
Education	○	○	○	○	○	○	○	○	○	○

Q2.4 How satisfied are you with your cardiac rehabilitation education?

	Extremely Dissatisfied	Dissatisfied	Satisfied	Extremely Satisfied
Delivery of education	○	○	○	○
Personalization of education	○	○	○	○
Clarity of information	○	○	○	○

End of Block: Satisfaction

Start of Block: VR Only

Display This Question:

If During what session did you complete cardiac rehabilitation? = 8 am

Or During what session did you complete cardiac rehabilitation? = 10 am

Or During what session did you complete cardiac rehabilitation? = 1:30 pm

Q3.1 Because of the virtual reality walking trails...

	Disagree	Undecided	Agree
I looked forward to treatment sessions more.	○	○	○
I enjoyed my treatment sessions more.	○	○	○
I felt more engaged in my treatment.	○	○	○
I wanted to spend longer on the treadmill.	○	○	○
I gained a better understanding of my cardiac health.	○	○	○
I did not see any impact on my treatment.	○	○	○
I was unwantedly distracted from my treatment.	○	○	○
I did not enjoy how I received my education.	○	○	○
I felt isolated from the rest of my class.	○	○	○
I dreaded my time on the treadmill.	○	○	○

Display This Question:

If During what session did you complete cardiac rehabilitation? = 8 am

Or During what session did you complete cardiac rehabilitation? = 10 am

Or During what session did you complete cardiac rehabilitation? = 1:30 pm

Q3.2 Please use this space to express how the virtual reality walking trails affected your cardiac rehabilitation experience.

____

____

____

____

____

End of Block: VR Only

Start of Block: Understanding Education

Q4.1 How often should you exercise?

- ○ Whenever you feel like it
- ○ At least once per week for 20 minutes
- ○ Three days per week for 10-15 minutes
- ○ Five or more days per week for 30-60 minutes
- ○ I don't know

Q4.2 What are the most important things to consider when grocery shopping for heart healthy foods?

- ○ Low fat and low sodium foods
- ○ Low calorie and low carb foods
- ○ Low sugar and high fiber foods
- ○ I don't know

Q4.3 How often should you take your blood pressure medication?

- When you remember
- When you are experiencing symptoms
- As often as directed by your doctor, usually once per day
- I don't know

Q4.4 What are the symptoms of heart failure?

- Shortness of breath
- Swelling of legs and ankles
- Weight gain
- All of the above
- None of the above
- I don't know

Q4.5 What type of medicine is used to control your cholesterol?

- ◯ A statin, such as atorvastatin (Lipitor)
- ◯ A beta blocker, such as metoprolol (Lopressor)
- ◯ An ACE inhibitor, such as lisinopril (Zestril)
- ◯ I don't know

End of Block: Understanding Education

Start of Block: Additional Comments

Q5.1 Please use this space for any additional comments regarding your experience with cardiac rehabilitation and our study.

____

____

____

____

____

End of Block: Additional Comments
