# Supplementary figures and images for: Effect of a Virtual Reality–Enhanced Exercise and Education Intervention on Patient Engagement and Learning in Cardiac Rehabilitation: Randomized Controlled Trial
Source: J Med Internet Res. 2021 Apr 15;23(4):e23882. doi: 10.2196/23882 (PMC8085751; doi:10.2196/23882)

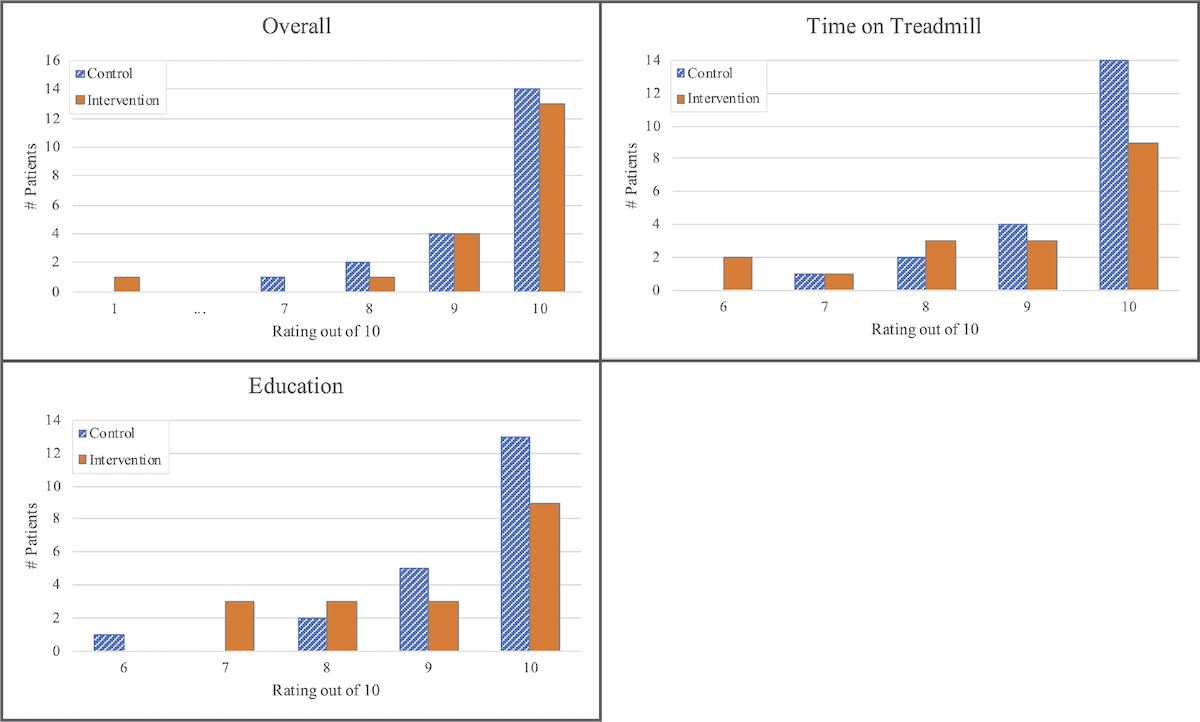

Supplement: Multimedia Appendix 4 [file jmir_v23i4e23882_app4.png]
